# Supplementary material for: Mechanoreceptive Aβ primary afferents discriminate naturalistic social touch inputs at a functionally relevant time scale
Source: ArXiv. 2025 Mar 12:arXiv:2503.09672v1. Preprint. [Version 1] (PMC11952579)
Supplement: Supplement 1 [file NIHPP2503.09672v1-supplement-1.pdf]

A

Partial η^2 Effect Sizes for Fig. 3A, 3B

Mean IFF	SA-II	HFA	Field	CT	FA-II	MS
Tapping - Stroking	0.52	0.53	0.00059	0.24	0.33	0.23
Stroking - Holding	0.66	0.8	0.36	0.52	0.32	0.23
Tapping - Holding	0.72	0.47	0.56	0.048	6.1E-5	0.12

# of Spikes	SA-II	HFA	Field	CT	FA-II	MS
Tapping - Stroking	0.51	0.68	0.4	1.1E-5	0.32	0.32
Stroking - Holding	0.6	0.27	0.078	0.32	0.42	0.0022
Tapping - Holding	3.9E-5	0.75	0.39	0.36	0.18	0.38

B

LMEM Test Results and Partial η^2 Effect Sizes for Fig. 3C

Tapping	SA-II	HFA	Field	CT	FA-II	MS
SA-II		-<br>0.12	-<br>0.026	*<br>0.37	-<br>8.8 E-5	****<br>0.7
HFA			-<br>0.017	***<br>0.52	-<br>0.043	****<br>0.75
Field				*<br>0.37	-<br>0.0097	***<br>0.57
CT					-<br>0.20	-<br>0.0069
FA-II						**<br>0.32

Stroking	SA-II	HFA	Field	CT	FA-II	MS
SA-II		-<br>0.09	-<br>0.07	***<br>0.68	**<br>0.6	****<br>0.76
HFA			-<br>0.0018	***<br>0.63	***<br>0.57	****<br>0.71
Field				**<br>0.47	*<br>0.42	***<br>0.55
CT					-<br>0.066	***<br>0.58
FA-II						***<br>0.6

Holding	SA-II	HFA	Field	CT	FA-II	MS
SA-II		*<br>0.34	*<br>0.47	**<br>0.54	**<br>0.71	****<br>0.75
HFA			-<br>0.0098	-<br>0.056	*<br>0.31	**<br>0.36
Field				-<br>0.043	**<br>0.49	***<br>0.5
CT					*<br>0.46	**<br>0.43
FA-II						-<br>0.012

Figure S1. (A) Partial η^2 effect sizes for the LMEM tests reported in Figure 3A, 3B. (B) LMEM test results (upper line in each cell) and the corresponding partial η^2 effect sizes (bottom line in each cell) for the pairwise comparison reported in Figure 3C. *$p < 0.05$, **$p < 0.01$, ***$p < 0.001$, ****$p < 0.0001$.

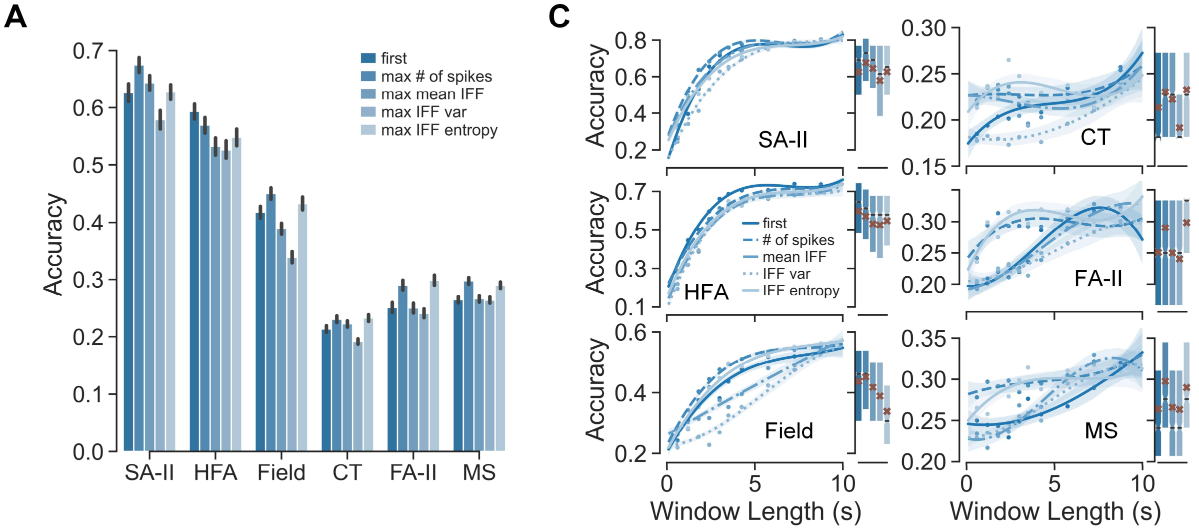

B

SA-II ****	first	count	mean	var	entropy
first	0.63	***	-	****	-
count		0.67	***	****	****
mean			0.64	****	-
var				0.58	***
entropy					0.63

HFA ****	first	count	mean	var	entropy
first	0.59	**	****	****	****
count		0.57	**	***	-
mean			0.53	-	-
var				0.53	-
entropy					0.55

Field ****	first	count	mean	var	entropy
first	0.42	****	***	****	-
count		0.45	****	****	*
mean			0.39	****	****
var				0.34	****
entropy					0.43

CT ****	first	count	mean	var	entropy
first	0.21	***	*	****	****
count		0.23	-	****	-
mean			0.22	****	-
var				0.19	****
entropy					0.23

FA-II ****	first	count	mean	var	entropy
first	0.25	****	-	-	****
count		0.29	****	****	-
mean			0.25	-	****
var				0.24	****
entropy					0.30

MS ****	first	count	mean	var	entropy
first	0.26	****	-	-	****
#		0.30	****	****	-
mean			0.27	-	****
var				0.26	****
entropy					0.29

Figure S2. (A) Classification accuracies across window position metrics averaged over all window lengths for each afferent subtype. (B) *$p < 0.05$, **$p < 0.01$, ***$p < 0.001$, ****$p < 0.0001$ were derived by Mann–Whitney U tests with Benjamini-Hochberg post-hoc correction. (C) Classification accuracies across window position metrics along with the increase of window length. Curves were fitted using third-order polynomial functions, points denote means of 10 evenly-binned data. Bar plots show distributions of classification accuracies over all window lengths per window position metric, and brown cross markers denote means per window position metric.
